# Smoking attitudes, self-reported practices, and COPD knowledge among adults aged 20–59 years: Insights from a Japanese sample

**DOI:** 10.18332/tid/200855

**Published:** 2025-03-28

**Authors:** Yolanda De Fatima De Oliveira Graca, Liu Yang, Cui Mingyu, Afsari Banu Alpona, Taeko Watanabe, Yuko Sawada, Emiko Tanaka, Tokie Anme

**Affiliations:** 1 Graduate School of Comprehensive Human Sciences, University of Tsukuba, Tsukuba, Japan; 2 Department of Nursing and Nutrition, Shukutoku University, Chiba, Japan; 3 Department of Physical Therapy, Morinomiya University of Medical Sciences, Osaka, Japan; 4 Faculty of Nursing, Musashino University, Tokyo, Japan; 5 Faculty of Medicine, University of Tsukuba, Tsukuba, Japan

**Keywords:** smoking attitudes, self-reported practices, COPD knowledge, Japan

## Abstract

**INTRODUCTION:**

Smoking remains a significant public health issue globally, despite efforts to reduce tobacco use. In Japan, smoking persists, particularly among certain groups.

**METHODS:**

This study investigates smoking attitudes, self-reported practices, and COPD knowledge among Japanese adults aged 20–59 years to identify intervention targets for reducing smoking prevalence and improving public health. Data from the 2020 ‘Community Empowerment and Well-Being and Healthy Long-term Care: Evidence from a Cohort Study (CEC)’ project were analyzed, including 537 participants categorized as smokers, former smokers, or non-smokers.

**RESULTS:**

This study shows that gender (male) is a strong predictor of current smoking across all age groups. Individuals with higher smoking awareness are less likely to smoke, especially in older adults. While COPD awareness is significantly associated with smoking only in the 20–29 age group. Among smokers who attempted to quit, 27.7% expressed willingness to quit, but only 3.0% were interested in cessation programs.

**CONCLUSIONS:**

A proportion of participants had a smoking history, highlighting tobacco use prevalence. Despite widespread support for anti-smoking measures, a disconnection between attitudes and behaviors persists. While most participants had heard of COPD, deeper knowledge of the disease and its symptoms was limited. These findings emphasize the need for anti-smoking policies targeting educational interventions and improving COPD awareness to promote behavior change and reduce smoking prevalence.

## INTRODUCTION

Tobacco smoking is one of the leading preventable causes of morbidity and mortality worldwide, contributing to a wide range of chronic diseases, including cardiovascular diseases, cancers, and respiratory conditions such as chronic obstructive pulmonary disease (COPD)^1^.

In Japan, where cultural attitudes towards smoking and health may differ from western contexts, it is essential to consider these factors when interpreting smoking behaviors and attitudes^2^. Smoking has undergone significant changes in Japan over the past few decades^3,4^. While overall smoking rates have declined, challenges persist in addressing smoking-related issues across various population sections^4^. The incidence of smoking is significant, particularly among men, with around 38% of men and 11% of women aged >20 years identified as smokers. This contributes to a significant health burden, particularly for Japanese men. It is crucial to consider these cultural factors when analyzing smoking behaviors and attitudes in Japan^5^.

Research suggests that knowledge of the health risks associated with smoking plays a crucial role in shaping smoking behaviors and attitudes. Understanding smoking attitudes, self-reported practices, and knowledge about chronic obstructive pulmonary disease (COPD) among adults aged 20–59 years is vital for developing effective public health strategies. Higher education level is linked to increased awareness of smoking-related health hazards, influencing smoking practices and intentions to quit^6,7^. This implies that educational interventions could be instrumental in enhancing knowledge and modifying smoking behaviors among young adults^7^.

Self-reported smoking practices are influenced by a complex interplay of knowledge, attitudes, and social factors. Studies have shown that individuals may acknowledge the health risks of smoking but often underestimate their personal vulnerability, particularly in younger populations^8,9^. This disconnection can lead to continued smoking despite awareness of its dangers. Furthermore, social context, including peer influences and societal norms, significantly impacts smoking behaviors. Young adults who perceive smoking as socially acceptable are less likely to quit, even if they are aware of its health risks^10,11^.

The relationship between smoking knowledge and attitudes towards COPD is particularly noteworthy. Knowledge about COPD and its association with smoking is often limited among smokers, hindering cessation efforts. Research indicates that many smokers are unaware of the specific health consequences of smoking, such as its role in developing COPD, which can contribute to continued smoking habits^12,13^. Moreover, educational interventions targeting smoking cessation have been shown to improve knowledge about smoking-related diseases, including COPD, fostering more positive attitudes towards quitting^14^.

Chronic obstructive pulmonary disease (COPD) is a significant public health issue, which is commonly diagnosed after the age of 40 years; however, individuals can be diagnosed as early as in their 20s or 30s, particularly in populations with high smoking prevalence. Smoking is the primary risk factor for COPD, and many individuals with the disease often do not recognize smoking as the main cause of their condition, complicating smoking cessation efforts^15^. This disconnection can be attributed to a self-judgmental attitude that hampers acceptance of the diagnosis and compliance with medical advice to quit smoking^16^. Early interventions in this population can significantly impact future health outcomes and reduce the burden of COPD on the healthcare system. Healthcare professionals’ attitudes towards smoking cessation play a critical role; doctors and healthcare professional supportive approach is linked to higher rates of smoking cessation among patients^17^. Investigating the factors influencing self-reported practices can lead to early detection. This study aims to inform the development of targeted public health interventions to promote smoking cessation and encourage earlier identification and awareness of COPD in Japan.

By addressing both knowledge deficiencies and social factors like stigma, educational interventions can significantly increase smoking cessation rates and decrease the incidence of smoking-related illnesses, including COPD. This is especially crucial for adults aged 20–59 years, who may face stigmatization that can negatively affect their mental health and, consequently, their smoking habits. Research has demonstrated that individuals with a longer smoking history may internalize stigma and self-blame, hindering their willingness to seek help from cessation programs^18^. This study seeks to evaluate attitudes towards smoking, understanding of COPD, and self-reported behaviors among Japanese adults aged 20–59 years.

## METHODS

This cross-sectional study was conducted in 2020 in T Village, Aichi Prefecture, Japan, a thriving agricultural community with approximately 4800 residents. Participants were recruited from the ‘Community Empowerment and Care for Well-being and Healthy Longevity: Evidence from a Cohort Study (CEC)’, a program initiated in 1991. The CEC is a cohort study conducted every 2 to 3 years among residents in the suburbs of central Japan, focusing on factors related to well-being and longevity. A self-reported questionnaire was administered to a target population of 692 participants. After excluding 38 participants due to missing demographic data (e.g. age and gender), the baseline sample consisted of 654 participants. Further exclusions were made for 117 participants with incomplete data on smoking attitudes, self-reported practices, and COPD knowledge, resulting in a final sample size of 537 participants for analysis. The study included individuals aged 20–59 years, encompassing current smokers, former smokers, and never smokers. Participants with a diagnosed history of COPD or other respiratory illnesses, as well as those with incomplete or missing data, were excluded from the analysis.

### Ethical considerations

This study received approval from the Ethics Committee of the University of Tsukuba (No. 1331-5). The data were provided by the municipality through formal agreements and was anonymized. In compliance with ethical guidelines, the research data were managed by the individual responsible for information management. Details about the project were publicly announced and posted on the local government’s website, allowing residents to opt out of the study if they wished.

### Smoking attitudes

Smoking attitudes were assessed using a self-reported questionnaire adapted from validated measures^19-21^. Participants were asked to indicate their agreement with the following statements using a yes/no response format: ‘I think cigarettes are good’, ‘I will never quit smoking’, ‘I get angry when people advise me to quit smoking’, ‘I get angry when the mass media talks about the harms of smoking’, and ‘I believe measures against smoking are necessary’. A ‘yes’ response was considered positive and a ‘no’ response was considered negative.

### Self-reported practices

Self-reported practices were assessed using a self-reported questionnaire using closed-ended question (yes or no) that captured information on smoking cessation attempts, participation in smoking cessation programs, and engagement in health and nutrition events. Participants were asked about their desire to quit smoking, previous attempts to quit, and willingness to participate in smoking cessation programs. They were also asked about their participation in health and nutrition events and lifestyle-related disease events.

### COPD knowledge

COPD knowledge was assessed using a self-reported questionnaire with yes/no response options. ‘Yes’ responses were considered positive, and ‘no’ responses negative. The questionnaire explored participants’ awareness of COPD, their perceptions of its causes (specifically focusing on risk factors, particularly smoking), and their recognition of common symptoms (coughing, shortness of breath, and wheezing). Participants were specifically asked about: awareness of COPD, ‘Do you know that there is a disease called COPD (chronic obstructive pulmonary disease)?’; perceived causes of COPD, ‘What do you think are the causes of COPD?’; and recognized symptoms of COPD: ‘What symptoms do you think COPD has?’.

### Covariates

Covariates included age (categorized into 20–29, 30–39, 40–49, and 50–59 years), gender, (male and female), regular breakfast intake (yes, no), long-term care (receive, don’t receive), alcohol consumption (drink, don’t drink), exercise (yes, no), and smoking history (cigarettes per day and smoking years). Smoking status was assessed using a 3-point Likert scale (current smoker, former smoker, never smokers).

### Statistical analysis

Descriptive statistics, including frequencies and percentages, were calculated to summarize the characteristics of the study sample. Chi-squared tests or Fisher’s exact tests (where appropriate) were used to explore relationships between categorical variables. Logistic regression models were used to assess the associations between the variables of interest, with results expressed as odds ratios and 95% confidence intervals. The adjustment was made using a covariate adjustment method, controlling for the following factors: gender (male as reference ?), smoking status, COPD symptoms, COPD cause, COPD awareness, and smoking awareness leading to the calculation of AOR and 95% confidence intervals (95% CI). All statistical analyses were conducted using SPSS version 28. A p<0.05 was considered to indicate statistical significance.

## RESULTS

The demographic characteristics of the study participants in Table 1, reveal a predominantly middle-aged group, with the majority aged 40–49 years (37.1%), followed by 30–39 years (21.8%) and 50–59 years (24.4%). The sample consisted of 44.7% males and 55.3% females. Only 0.9% of participants reported receiving long-term care, while 99.1% did not. Alcohol consumption was reported by 46.0% of participants, and 92.7% reported regularly eating breakfast. Exercise habits were nearly evenly split, with 48.4% exercising frequently and 51.6% rarely exercising. Regarding smoking status, (12.3%) were current smokers, (15.6%) were ex-smokers, and (72.1%) were never smokers.

**Table 1:** Demographic characteristics of the participants, smoking attitudes and measures, Achi prefecture, Japan, 2020 (N=537)

*Characteristics*	*Categories*	*n*	*%*
**Age** (years)	20–29	90	16.8
	30–39	117	21.8
	40–49	199	37.1
	50–59	131	24.4
**Gender**	Male	240	44.7
	Female	297	55.3
**Long-term care**	Yes	5	0.9
	No	532	99.1
**Alcohol consumption**	Almost everyday	247	46.0
	Don’t drink	290	54.0
**Regular breakfast intake**	Always	498	92.7
	Hardly eats	39	7.3
**Exercise**	Frequently	260	48.4
	Rarely	277	51.6
**Smoking history**	Current smoker	66	12.3
	Former smoker	84	15.6
	Never smoker	387	72.1
**Duration of smoking** (years)	0	471	87.7
	1–10	23	5.2
	11–19	8	2.4
	20–29	28	5.2
	30–39	15	2.8
	≥40	2	0.4
**Cigarettes/day**	0	471	87.7
	1–10	28	5.2
	11–20	9	1.7
	21–30	23	4.3
	31–40	3	0.6
	≥41	3	0.6
**Measures toward smoking**	Yes	420	78.2
	No	117	21.8
**Attempt to quit smoking**	Yes	15	2.8
	No	522	97.2
**Attempt to quit but cannot**	Yes	29	5.4
	No	508	94.6
**Attempt to smoke less**	Yes	18	3.4
	No	519	96.6
**Participate in cessation programs**	Yes	2	0.4
	No	535	99.6
**Think cigarettes are good**	Yes	2	0.7
	No	533	99.3
**Angry at advice to quit**	Yes	4	0.7
	No	533	99.3
**Angry at mass media talks of harm of smoking**	Yes	4	0.7
	No	533	99.3
**Never quit smoking**	Yes	9	1.7
	No	528	98.3

The participants COPD knowledge overall was: 17.1% were familiar with COPD, 39.1% had heard of it but lacked knowledge, and 43.8% were not familiar with the disease. As detailed in Table 2, awareness of COPD symptoms was low, with only 52.7% recognizing shortness of breath, 44.5% recognizing coughing, and 34.3% recognizing phlegm as symptoms. A significant proportion of participants (41.3%) admitted they did not know the symptoms of COPD. While (68.3%) correctly identified tobacco use as a cause of COPD, but awareness of other causes, such as dust and chemicals (34.8%), was lower. Finally, regarding participation in health and nutrition workshops, it was reported by 24.6% that they had attended, while 75.4% did not participate.

**Table 2:** Participants COPD knowledge and selfreported practices, Achi prefecture, Japan (N=537)

	*Categories*	*n*	*%*
**COPD knowledge**	Familiar	92	17.1
	Heard of, but don’t know	210	39.1
	Not familiar	235	43.8
**Knowledge of COPD symptoms**			
Coughing	Yes	239	44.5
	No	298	55.5
Phlegm	Yes	184	34.3
	No	353	65.7
Shortness of breath	Yes	283	52.7
	No	254	47.3
Don’t know	Yes	222	41.3
	No	315	58.7
**Probable cause of COPD**			
Eating habits	Yes	79	14.7
	No	458	85.3
Exercise	Yes	86	16.0
	No	451	84.0
Tobacco (smoking and passive smoking)	Yes	367	68.3
	No	170	31.7
Dust and chemicals	Yes	187	34.8
	No	350	65.2
Other	Yes	4	0.7
	No	533	99.3
Not sure	Yes	170	31.7
	No	367	68.3
Health and nutrient workshop participation	Yes	132	24.6
	No	405	75.4
Lifestyle-related disease workshop participation	Yes	76	14.2
	No	461	85.8

The analysis in Table 3 presents smoking status stratified by age, gender, and self-reported health practices. Males were significantly more likely to be current smokers (84.8%) compared to females (15.2%). This trend was consistent across all age groups (p<0.05). The largest proportion of current smokers were aged 40–49 years (45.5%), followed by 30–39 years (22.7%). Smoking status did not vary significantly by age (p=0.43). Participation by current smokers was less likely in health and nutrition workshops (9.1%) compared to ex-smokers (27.4%) and never smokers (26.6%). Similarly, participation in disease prevention workshops was low among current smokers (7.6%). Finally, attitudes towards smoking measures revealed that never smokers (81.9%) and former smokers (73.8%) demonstrated significantly greater support for such measures compared to current smokers (62.1%).

**Table 3:** Smoking status by age, gender, and self-reported practices, and smoking measures, as COPD knowledge and COPD symptoms of smoking predictors, Achi prefecture, Japan, 2020 (N=537)

*Variable*	*Categories*	*Current smoker* *(N=66)*		*Former smoker* *(N=84)*		*Never smoker* *(N=387)*			
		*n*	*%*	*n*	*%*	*n*	*%*	*χ^2^*	p*
**Gender**	Male	56	84.8	65	77.4	119	30.7	9.805	**0.01**
	Female	10	15.2	19	22.6	268	69.3		
**Age** (years)	20–29	7	10.6	6	7.1	77	19.9	2.953	0.43
	30–39	15	22.7	17	20.2	85	22.0		
	40–49	30	45.5	38	45.2	131	33.9		
	50–59	14	21.2	23	27.4	94	24.3		
**Health nutrient workshops**	Yes	6	9.1	23	27.4	103	26.6	9.762	**0.00**
	No	60	90.9	61	72.6	284	73.4		
**Disease prevention workshops**	Yes	5	7.6	16	19.0	55	14.2	4.007	0.13
	No	61	92.4	68	81.0	332	85.8		
**Measures toward smoking**	Yes	41	62.1	62	73.8	317	81.9	4.093	**0.01**
	No	25	37.9	22	26.2	70	18.1		
**COPD**	Familiar	12	18.2	13	15.5	67	17.3	0.512	0.972
	Not sure	24	36.4	35	41.7	151	39.0		
	Not Familiar	30	45.5	36	42.9	169	43.7		
**Symptoms Cough**	Yes	20	30.3	33	39.3	186	48.1	8.299	**0.016**
	No	46	69.7	51	60.7	201	51.9		
**Symptoms Phlegm**	Yes	15	22.7	21	25.0	148	38.2	9.821	**0.007**
	No	51	77.3	63	75.0	239	61.8		
**Symptoms Shortness of breath**	Yes	25	37.9	40	47.6	218	56.3	8.733	**0.013**
	No	41	62.1	44	52.4	169	43.7		
**Don’t know Symptoms**	Yes	35	53.0	39	46.4	148	38.2	6.147	**0.046**
	No	31	47.0	45	53.6	239	61.8		

* p<0.05 is significant.

For the logistic regression analysis, the results presented in Table 4, examined predictive factors for current smoking status. This revealed several factors significantly associated with current smoking status, males had significantly higher odds of being current smokers across all age groups (e.g. 20–29 years; AOR=1.34; 95% CI: 1.06–1.68, p<0.05). Smoking measures were associated with a reduced likelihood of being a current smoker (e.g. 20–29 years; AOR=0.74; 95% CI: 0.58–0.95, p<0.05). Smoking status did not vary significantly by age, though the age group of 40–49 years had the highest proportion of current smokers.

**Table 4:** Smoking status: Results from logistic regression models by age, Achi prefecture, Japan, 2020 (N=537)

*Age (years)*	*Variables*	*AOR*	*95% CI*	*p*
**20–29**	COPD symptoms	1.29	0.90–1.85	0.167
	COPD cause	1.00	0.70–1.42	0.986
	COPD awareness	1.20	1.01–1.42	**0.044**
	Smoking measures	0.74	0.58–0.95	**0.021**
	Gender	1.34	1.06–1.68	**0.016**
**30–39**	COPD symptoms	0.93	0.64–1.34	0.684
	COPD cause	1.07	0.74–1.54	0.720
	COPD awareness	1.04	0.88–1.25	0.630
	Smoking measures	1.02	0.78–1.34	0.859
	Gender	2.08	1.64–2.63	**0.000**
**40–49**	COPD symptoms	0.96	0.72–1.28	0.808
	COPD cause	1.06	0.81–1.40	0.660
	COPD awareness	1.15	0.97–1.35	0.101
	Smoking measures	0.78	0.62–0.99	**0.041**
	Gender	2.17	1.79–2.62	**0.000**
**50–59**	COPD symptoms	0.99	0.73–1.33	0.936
	COPD cause	1.34	0.99–1.82	0.065
	COPD awareness	1.13	0.95–1.35	0.176
	Smoking measures	0.64	0.48–0.84	**0.002**
	Gender	1.70	1.38–2.09	**0.000**

AOR: adjusted odds ratio. Models adjusted for gender, smoking status, COPD symptoms, COPD cause, COPD awareness, and smoking awareness. Bold p-values indicate statistical significance (p<0.05). COPD: chronic obstructive pulmonary disease.

Demographic characteristics of current smokers, given in Supplementary file Table 1, showed that among the 66 current smokers, the majority were male (84.8%), with only 15.2% female. The largest proportion of current smokers were aged 40–49 years (45.5%), followed by 30–39 years (22.7%). Most current smokers had been smoking for 20–29 years (42.4%), and the majority consumed 1–10 cigarettes per day (42.4%). A significant proportion of current smokers (62.1%) supported smoking measures, while 37.9% did not. Only 22.7% of current smokers reported attempting to quit, and 43.9% of those who tried were unable to quit.

## DISCUSSION

This study was conducted to observe the smoking attitudes self-reported practices and COPD knowledge among Japanese adults. A significant majority of participants, particularly never smokers and ex-smokers supported smoking control measures. This indicates a strong public willingness to adopt policies aimed at reducing smoking prevalence, which can be leveraged to strengthen public health campaigns. This corresponds with a study in New Zealand, where both smokers and non-smokers exhibit substantial support for initiatives such as smoke-free playgrounds and vehicles, with a widespread agreement on the objective of decreasing smoking prevalence to around 5% by 2025^22^. Our study found a clear gender disparity, with males being significantly more likely to be current smokers. This finding highlights the need for smoking cessation programs that could be tailored specifically for males. Females were significantly less likely to be current smokers, this can positively be reinforced through continued public health efforts to maintain low smoking rates among women. Seen in by several studies across different geographies for example in Vietnam, 45.3% of adult smokers were men, demonstrating a significant incidence of smoking among males especially in rural areas^23^. Tobacco consumption is the foremost global cause of preventable mortality, resulting in about 8 million fatalities annually. In this study, most participants correctly identified tobacco use as a leading cause of COPD^1^. This demonstrates a foundational level of awareness that can be built upon in public health messaging to emphasize the risks of smoking and tobacco use. Shortness of breath was identified as a symptom of COPD by more than half of the participants. Nevertheless, there is still a sizable section of the general public which is unfamiliar with COPD and educational campaigns to improve recognition of COPD symptoms, such as coughing and phlegm^24^. According to a study, younger smokers had a mean perceived risk score for COPD of 10.94, which suggests that they were not as conscious of the risks associated with the disease. Higher COPD awareness was linked to a marginally higher chance of being a current smoker among the different age groups in this study, specifically the 20–29 age group. This research shows that contrary to popular belief, younger smokers might not be completely aware of the dangers of COPD, highlighting the need for focused education to close this knowledge gap^25^. The consistent finding across age groups that supporting smoking control measures reduces the likelihood of smoking, underscores the importance of policies such as smoking bans, taxation, and public awareness campaigns.

### Strengths and limitations

This study, focusing on smoking attitudes, self-reported practices, and COPD knowledge among Japanese adults aged 20–59 years, presents several limitations. Firstly, the cross-sectional design precludes the establishment of causal relationships between variables. Secondly, the study’s focus on a specific age range and the Japanese population may limit the generalizability of the findings to other age groups and populations. Thirdly, the small sample size is a limitation, which may undermine the study power, compared to studies with longitudinal designs and larger sample sizes. Studies with longitudinal and larger sample sizes are needed. Fourthly, self-reported data on smoking behaviors and COPD knowledge may be subject to recall bias. While the relatively small sample size of female smokers is another limitation.

Despite these limitations, this study offers several strengths. Firstly, it provides valuable insights into the smoking-related behaviors and knowledge of a significant segment of the Japanese population. Secondly, the findings can inform the development of targeted health education campaigns and smoking cessation interventions tailored to this specific demographic.

To address these limitations, longitudinal studies to assess causality, studies with more diverse samples, or studies incorporating objective measures of smoking status are needed. Building upon the strengths of this study, future research can contribute significantly to a deeper understanding of smoking-related behaviors and the development of effective public health interventions to reduce the burden of smoking-related diseases in Japan and beyond.

## CONCLUSIONS

The findings of the present study reveal important patterns in smoking behaviors, awareness, and attitudes among the study participants. The low prevalence of attempts to quit smoking, despite broad support for anti-smoking measures, highlights the need for targeted interventions and improved cessation support. Additionally, the recognition of gender differences in health awareness underscores the importance of tailoring public health strategies to different demographic groups. This research can help to address the significant public health challenge of COPD in Japan and promote healthier lifestyles.

## Supplementary Material



## Data Availability

The data supporting this research are available from the authors on reasonable request.
