# Supplementary material for: Smoking attitudes, self-reported practices, and COPD knowledge among adults aged 20–59 years: Insights from a Japanese sample
Source: Tob Induc Dis. 2025 Mar 28;23:10.18332/tid/200855. doi: 10.18332/tid/200855 (PMC11951969; doi:10.18332/tid/200855)
Supplement: Supplementary file 1 [file TID-23-42-s1.pdf]

Supplementary file Table 1: Demographic characteristics of current smokers of Achi prefecture, Japan, 2020 (N=66)

Variables	Categories	(n)	(%)
Age(years)	20-29	7	10.6
	30-39	15	22.7
	40-49	30	45.5
	50-59	14	21.2
Gender	Male	56	84.8
	Female	10	15.2
Duration of smoking (yrs)	1-10	13	19.7
	11-19	8	12.1
	20-29	28	42.4
	30-39	15	22.7
	≥40	2	3.0
Number of cigarettes/days	1-10	28	42.4
	11-20	9	13.6
	21-30	23	34.8
	31-40	3	4.5
	≥41	3	4.5
Measures toward smoking	Yes	41	62.1
	No	25	37.9
Attempt to quit smoking	Yes	15	22.7
	No	51	77.3
Attempt to quit but can't	Yes	29	43.9
	No	37	56.1
Attempt to smoke less	Yes	18	27.3
	No	48	72.7
Participate in cessation programs	Yes	2	3.0
	No	64	97.0
Think cigarettes are good	Yes	2	3.0
	No	64	97.0
Angry at advice to quit	Yes	4	6.1
	No	62	93.9

Angry at mass media talks of harm of smoking	Yes	4	6.1
	No	62	93.9
Never quit smoking	Yes	9	13.6
	No	57	86.4

© 2025 Graca Y.D.F.F.O. et al.
